# Current Challenges in Providing Good Leukapheresis Products for Manufacturing of CAR-T Cells for Patients with Relapsed/Refractory NHL or ALL

**DOI:** 10.3390/cells9051225

**Published:** 2020-05-15

**Authors:** Felix Korell, Sascha Laier, Sandra Sauer, Kaya Veelken, Hannah Hennemann, Maria-Luisa Schubert, Tim Sauer, Petra Pavel, Carsten Mueller-Tidow, Peter Dreger, Michael Schmitt, Anita Schmitt

**Affiliations:** 1 Department of Internal Medicine V, University Hospital Heidelberg, 69120 Heidelberg, Germany; sandra.sauer@med.uni-heidelberg.de (S.S.); kaya.veelken@med.uni-heidelberg.de (K.V.); hannah.hennemann@med.uni-heidelberg.de (H.H.); maria-luisa.schubert@med.uni-heidelberg.de (M.-L.S.); tim.sauer@med.uni-heidelberg.de (T.S.); Carsten.Mueller-Tidow@med.uni-heidelberg.de (C.M.-T.); peter.dreger@med.uni-heidelberg.de (P.D.); michael.schmitt@med.uni-heidelberg.de (M.S.); anita.schmitt@med.uni-heidelberg.de (A.S.); 2 Institute of Clinical Transfusion Medicine and Cell Therapy (IKTZ), 89081 Heidelberg, Germany; sascha.laier@iktz-hd.de (S.L.); petra.pavel@iktz-hd.de (P.P.)

**Keywords:** CAR T cell, apheresis, lymphocyte collection, CD3 positive lymphocytes

## Abstract

Background: T lymphocyte collection through leukapheresis is an essential step for chimeric antigen receptor T (CAR-T) cell therapy. Timing of apheresis is challenging in heavily pretreated patients who suffer from rapid progressive disease and receive T cell impairing medication. Methods: A total of 75 unstimulated leukaphereses were analyzed including 45 aphereses in patients and 30 in healthy donors. Thereof, 41 adult patients with Non-Hodgkin’s lymphoma (85%) or acute lymphoblastic leukemia (15%) underwent leukapheresis for CAR-T cell production. Results: Sufficient lymphocytes were harvested from all patients even from those with low peripheral lymphocyte counts of 0.18/nL. Only four patients required a second leukapheresis session. Leukapheresis products contained a median of 98 × 10^8^ (9 - 341 × 10^8^) total nucleated cells (TNC) with 38 × 10^8^ (4 - 232 × 10^8^) CD3+ T cells. Leukapheresis products from healthy donors as well as from patients in complete remission were characterized by high TNC and CD3+ T lymphocyte counts. CAR-T cell products could be manufactured for all but one patient. Conclusions: Sufficient yield of lymphocytes for CAR-T cell production is feasible also for patients with low peripheral blood counts. Up to 12–15 L blood volume should be processed in patients with absolute lymphocyte counts ≤ 1.0/nL.

## 1. Introduction

T cells transduced with a chimeric antigen receptor (CAR) directed against CD19 have shown promising efficacy in patients with relapsed or refractory (r/r) B-lineage acute lymphoblastic leukemia (ALL) or r/r B-cell non-Hodgkin’s lymphoma (NHL) [1,2,3].

Currently, two CAR-T cell products are commercially available in Europe—tisagenlecleucel (Kymriah^®^) and axicabtagene ciloleucel (Axi-Cel; Yescarta^®^). Tisagenlecleucel is approved for r/r diffuse large B cell lymphoma (DLBCL) and for r/r B-ALL in children and young adolescents (≤25 years). Axicabtagene ciloleucel is approved for r/r DLBCL and primary mediastinal B cell lymphoma (PMBCL). 

A basic requirement for CAR-T cell manufacturing is a sufficient amount of T cells that have to be collected by an unstimulated leukapheresis. Thereafter, T cells are transduced with a retro- or lentiviral vector encoding the CAR. CAR-T cells are stimulated and expanded in cell culture, and eventually transfused back into the patient [4,5].

Some CAR-T cell products under current investigation are based on allogeneic T cells from healthy donors, while the commercial CAR-T products as well as the clinical study products mentioned here rely on autologous patient derived T cells. T cells from patients might be decreased in number or hampered by several lines of pretreatment and actual disease related treatment [6]. Furthermore, the timing of leukapheresis might constitute a problem for clinicians due to the progress of r/r disease in patients, thus urging for a therapeutic intervention or bridging [7].

Therefore we evaluated the following questions in this study: (i) what has to be checked before leukapheresis (i.e., inclusion/exclusion criteria), (ii) when has T cell impairing medication to be stopped before leukapheresis, (iii) can leukapheresis be performed in an out-patient setting, (iv) what problems will we face during leukapheresis of heavily pretreated patients in a r/r disease state, (v) is the lymphocyte collection in patients feasible and effective, (vi) how can we optimize the lymphocyte collection.

## 2. Materials and Methods

### 2.1. Patients and Healthy Donors

From September 2018 till November 2019, T cells were collected at our center from 41 patients via apheresis for subsequent CAR-T cell therapy after informed consent. Four patients received two separate apheresis sessions, resulting in a total of 45 apheresis sessions analyzed. Aphereses were performed to manufacture the following CAR-T cell products: axicabtagene ciloleucel (Kite-Gilead), tisagenlecleucel (Novartis, Basel, Switzerland), product for the clinical study HD-CAR-1 (University Hospital Heidelberg) (EudraCT: 2016-004808-60) [8]. For donor lymphocyte infusions T cells were collected from 30 healthy donors (HDs) after informed consent at our hospital in between 2014 and 2019.

### 2.2. Leukapheresis Procedures

Leukapheresis procedures were performed at the University Hospital Heidelberg in cooperation with the Institute of Clinical Transfusion Medicine and Cell Therapy (IKTZ, Heidelberg, Germany), Heidelberg, using Spectra Optia^®^ devices (Terumo, Tokyo, Japan). The apheresis lasted between 2 and 5 h with a 2-4 times total blood volume processed depending on the peripheral white blood cell and lymphocyte counts. ACD-A was used as anticoagulant in a blood to anticoagulant ratio of 12–15:1. All aphereses were performed over peripheral veins. In the case of difficult access of peripheral veins, ultrasound-guided puncture of peripheral veins was performed. For the commercial products a target total nucleated cell count of 20 × 10^8^ TNC and a T cell count of 10 × 10^8^ CD3+ T cells were aimed at. Table 1 highlights the different indications as well as the varying requirements and procedures set by the manufacturers regarding leukapheresis product, apheresis, and product application.

### 2.3. Leukapheresis Product

Samples of the apheresis product were analyzed by flow cytometry at the IKTZ. For viability analysis with propidium iodide and assessment of CD3+/CD45+ T cells and total nucleated cell count the MACsQuant-Analyzer 10^®^ (Miltenyi Biotech, Bergisch Gladbach, Germany) was used. Hematocrit and platelet count were determined with an automated hematology analyzer (XP-300^®^, Sysmex, Germany). Sterility of the leukapheresis product was proven using BACTEC™ flascs (Becton Dickinson, Franklin Lakes, NJ, USA) for automated blood culture testing (Hybeta, Germany) and BacT/ALERT^®^ (bioMérieux, Marcy-l’Étoile, France) at the blood donation center in Mannheim, Germany. For the tisagenlecleucel product the leukapheresis product was cryopreserved according to the specifications of the CAR-T cell manufacturer and picked up thereafter. For the axicabtagene ciloleucel product the leukapheresis product was picked up fresh on the same day of leukapheresis for centralized cryopreservation. For the HD-CAR-1 study the fresh leukapheresis product was transported to the GMP unit at the University Hospital Heidelberg for cryopreservation.

### 2.4. CAR-T Cell Products

Axicabtagene ciloleucel is an autologous anti-CD19 CAR-T cell product containing a second- generation CAR encoded by a retroviral vector with a single-chain variable fragment (scFv) targeting CD19 with CD3ζ and CD28 intracellular domains that signal T-cell activation. The CAR-T cells were applied to 22 r/r DLBCL patients and 1 r/r PMBCL patient in a total dose of 0.4 - 2 × 10^8^ CAR-T cells after lymphodepleting chemotherapy with fludarabine and cyclophosphamide [2]. Tisagenlecleucel is generated from autologous T cells transduced with a second-generation lentiviral vector to express an anti-CD19 CAR containing a CD3-zeta domain and a 4-1BB (CD137) domain as costimulatory signal. The CAR-T cells were applied to 2 r/r DLBCL patients in a total dose of 0.6 - 6 × 10^8^ CAR-T cells after lymphodepleting chemotherapy with fludarabine and cyclophosphamide [1]. In the HD-CAR-1 study autologous T cells were transduced with a third-generation retroviral CAR vector encoding for anti-CD19 with CD3ζ for T cell activation and CD28 and 4-1BB domains as costimulatory signals. The CAR-T cells were applied to r/r DLBCL, mantle cell lymphoma (MCL), follicular lymphoma (FL), chronic lymphocytic leukemia (CLL), and ALL patients in an escalating dose from 1 × 10^6^/m^2^, 5 × 10^6^/m^2^ up to 20 × 10^6^/m^2^ body surface area after lymphodepleting chemotherapy with fludarabine and cyclophosphamide [8].

### 2.5. Clinical Evaluation

Assessment of response to CAR-T cell therapy was defined as complete remission (CR), partial remission (PR), stable disease (SD), progressive disease (PD), and not yet evaluable (NE) according to Lugano standard response criteria for NHL [9] and with blasts-percentage and MRD testing for ALL [10].

### 2.6. Data Analysis

The data were analyzed by standard statistical measures, arithmetic median, and range (minimum/maximum). For the statistical analysis and data collection, Microsoft Excel^®^ was used. In addition, cut-off scores were calculated for apheresis product regarding CD3+ T cells and TNC counts. Calculations of significance were performed using IBM SPSS 20 for Windows (IBM Corp. Armonk, NY, USA). Differences in TNC and CD3+ T cell count were assessed by Student’s *t*-test. In all tests, a *p*-value < 0.05 was considered to be statistically significant.

## 3. Results

### 3.1. Patients Characteristics

**Figure 1:**
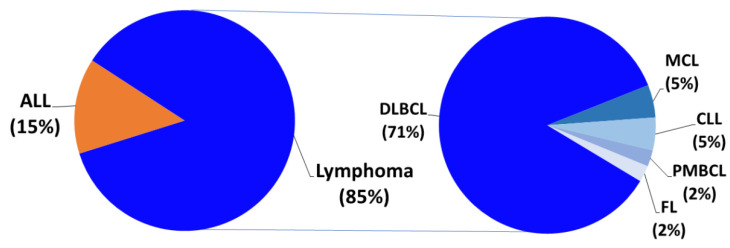
Distribution of patients undergoing leukapheresis by disease. The main share of patients had been diagnosed with lymphoma (85%), shown separately by subgroup: diffuse large B-cell lymphoma (DLBCL, 71%), mantle cell lymphoma (MCL, 5%), follicular lymphoma (FL, 2%), primary mediastinal B-cell lymphoma (PMBCL, 2%), or chronic lymphocytic leukemia (CLL, 5%). The other studied entity is the acute lymphoblastic leukemia (ALL, 15%).

Of the 41 patients included in this study, 29 were male and 12 were female. A total of 35 patients (85%) were diagnosed with lymphoma and six patients (15%) with ALL. Of all lymphoma patients, 29 patients (71%) had DLBCL, two (5%) had MCL, two (5%) had CLL, one (2%) had FL, and one (2%) had PMBCL. Figure 1 shows the distribution of the disease entities of the patients included in this study.

### 3.2. Baseline Analysis

The age and gender ratio of 25 patients receiving leukapheresis for axicabtagene ciloleucel, as displayed in Table 2, was comparable to epidemiological data on DLBCL and PMBCL [11,12]. Two patients received tisagenlecleucel, while 14 patients were given a product for the clinical study HD-CAR-1.

**Figure 2:**
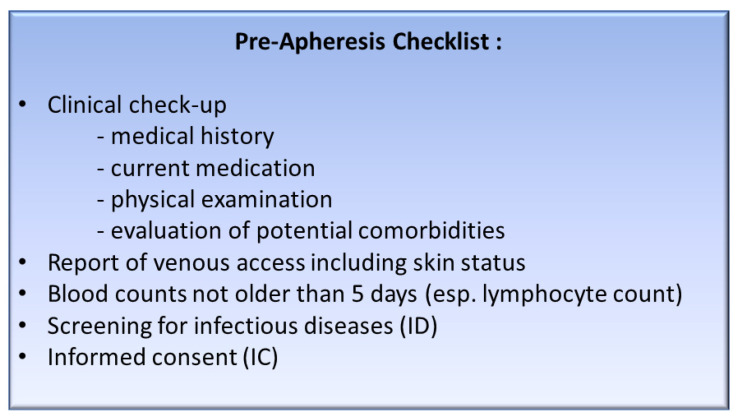
Checklist for requirements prior to leukapheresis. This figure displays an operational sequence description, analog to autologous or allogeneic cell therapy, as performed at the University Hospital of Heidelberg for patients prior to CAR-T cell apheresis.

**Figure 3:**
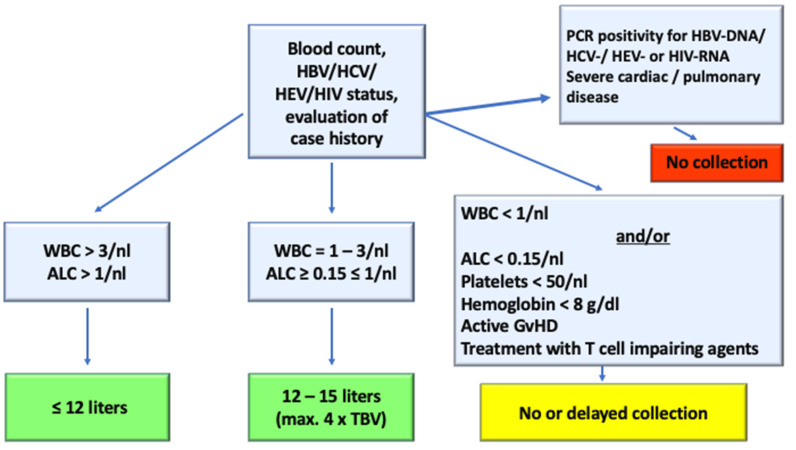
Apheresis algorithm towards CAR-T cell products. To increase the chance of a successful collection amount, an algorithm based on the leukocyte and lymphocyte count was created with additional listing of exclusion criteria leading to a possible cancellation or delayed collection. WBC = white blood cell count, ALC = absolute lymphocyte count, TBV = total blood volume.

Before leukapheresis a clinical check-up with screening for infectious disease markers, immunophenotyping by flow cytometry for CD4+ and CD19+ cells and differential blood count has to be performed for all patients (Figure 2). According to our algorithm (Figure 3), all 41 patients met the following inclusion criteria and therefore qualified for leukapheresis: hemoglobin > 8 g/dl, platelets > 50/nL, and white blood cell count > 1/nL. In addition, PCR revealed negative screening parameters for HBV, HCV, HEV, and HIV in all patients. Only patients with no signs of active GvHD, no florid infection, as well as no severe impairment of cardiac or pulmonary function were admitted to leukapheresis for CAR-T cell therapy. Patients evaluated in this study did not present a leukemic phase.

### 3.3. Apheresis Conditions

Leukapheresis was feasible in all patients and could be performed through peripheral venous access using the Spectra Optia™ device without any serious side effects. 

A total of 45 leukaphereses were performed in 41 patients. Four male patients required a second apheresis. These four patients received a median of four (2-5) prior therapies and were not more intensely pretreated when compared to all other evaluated patients with a median of five (2-8) prior therapies. These prior therapies were mostly rituximab based, with an allogeneic stem-cell transplantation in one patient three years before CAR-T cell therapy. For one of these patients, the initial manufacture of the CAR-T cell product failed due to infectious contamination of the first leukapheresis product potentially caused by a severe urinary tract infection that eventually led to a systemic antibiotic treatment of the patient. For the other three necessary repetitions of leukapheresis, the CAR-T cell products did not meet the specified release criteria. In two patients a particle of unknown origin was detected during CAR-T cell manufacturing process and in one patient, a second leukapheresis was necessary to obtain the required number of CAR transduced T cells. For three of the four patients that underwent second apheresis, a CAR-T cell product could be successfully manufactured. CAR-T cell production for one patient eventually failed due to a low number of functional CAR-T cells.

Main apheresis characteristics are shown in Table 3. A median blood volume of 12.0 L (5.8–15.0 L) with 2.4 times (1.2–3.9 times) total blood volume processed was handled. The median time of duration of the apheresis procedure was 240 min (120–300 min).

### 3.4. Apheresis Concentrate Characteristics

The average leukapheresis product contained a median of 98 × 10^8^ (9 - 341 × 10^8^) TNC including 38 × 10^8^ (4 - 232 × 10^8^) CD3+ T cells with a viability of 99.9% (99.6–100%) in a median volume of 237 mL (136–310 mL) with a hematocrit of 2.6% (1.1–7.4%) (Table 4). Total nucleated cell counts and total CD3+ T cell counts were highest in the lymphoma subgroup.

### 3.5. CAR-T Cell Manufacturing

For 40 of 41 patients, CAR-T cell production reached the release criteria of 0.4 - 2 × 10^8^ CAR T cells for axicabtagene ciloleucel, 0.6 - 6 × 10^8^ CAR-T cells for tisagenlecleucel, and dose escalation step III with 20 × 10^6^/m^2^ body surface for HD-CAR-1. For one patient, no sufficiently functional CAR-T cell product could be produced due to a particle of unknown origin in the first manufacturing and due to insufficient CAR-T cell function in the second manufacturing process. This patient received rituximab (R) with cisplatin in combination with high-dose cytarabine and dexamethasone (DHAP) 8 weeks prior to the first leukapheresis and 2 weeks before the second apheresis he received steroids and two times an irradiation of the thoracic spine (Table 5). One patient died due to disease progression before the anticipated day of CAR-T cell infusion, therefore a total of 39 patients received CAR-T cells.

### 3.6. Remission Status Obtained in the Patient

**Figure 4:**
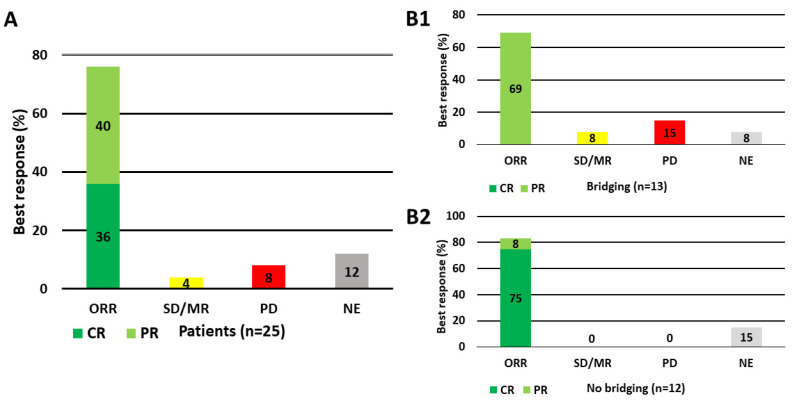
Best response (%) in patients receiving leukapheresis for axicabtagen ciloleucel. A total of 25 patients, all receiving leukapheresis for axicabtagene ciloleucel, were investigated for response (CR = complete response, PR = partial response, SD/MR = stable disease/mixed response, PD = progressive disease, NE = not evaluable). In 12% of patients, no remission data was available, either due to death of progressive disease prior to staging or not yet processed staging CT scans. (**A**) = all patients, (**B**) = patients requiring bridging (**B1**, upper panel) vs. patients requiring no bridging (**B2**, lower panel).

Of the 39 patients receiving a CAR-T cell product, data on outcome of 25 patients receiving leukapheresis for the Yescarta^®^ CAR-T cell product was available (Figure 4). The overall response rate (ORR) was 76% (36% complete and 40% partial remission). A total of 4% of patients had stable disease or mixed response, while progressive disease was seen in 8%. In 12% of patients, no remission data was available, either due to not yet processed staging CT scans or to death of progressive disease prior to staging. Results of the remaining patients, who were treated within ongoing clinical trials, will be reported separately.

### 3.7. Pre-Apheresis Lymphocyte Count and CD3+ Cell Yield by Response

**Figure 5:**
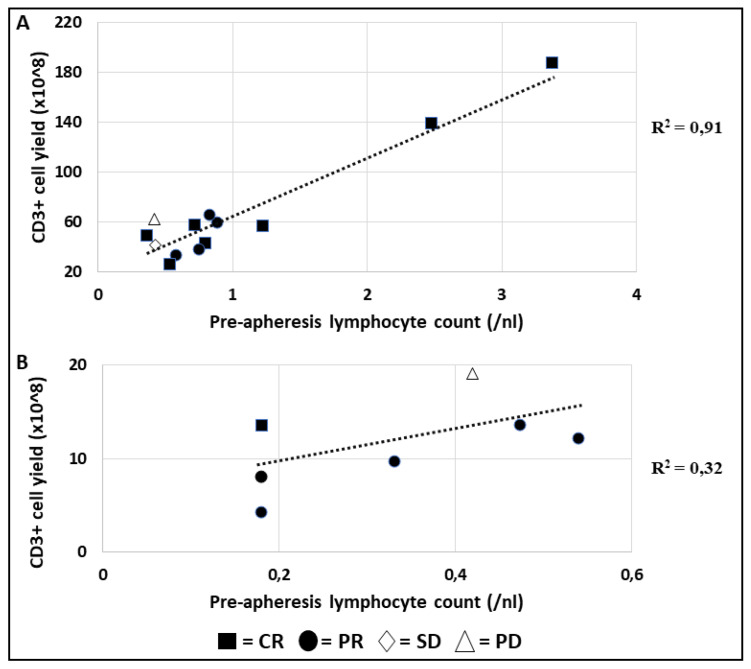
Overview pre-leukapheresis lymphocyte count and CD3+ cell yield by response (axicabtagen ciloleucel). In this figure, pre-apheresis data on lymphocyte count and CD3+ cell yield by response are shown. Group (**A**) displays a CD3+ cell yield of >20 (×10^8^), ((**B**) group ≤ 20 (×10^8^)). Splitting of this figure in two panel groups was done for improved graphic display.

Leukapheresis products of patients with known remission status were analyzed for lymphocyte count prior to apheresis and CD3+ T cell yield in the product (Figure 5). Three groups of patients could be defined according to their lymphocyte count in the peripheral blood prior to leukapheresis: patients with (1) a lymphocyte count of >1/nL, (2) a lymphocyte count between 0.6/nL and 1/nL and (3) a low lymphocyte count of <0.6/nL. All three patients with a lymphocyte count of >1/nL prior to apheresis reached a complete remission after CAR-T cell transfusion. In the subgroup of patients with a low lymphocyte count of ≥0.18/nL and <0.6/nL in the peripheral blood and a CD3+ cell yield of ≥4.2 × 10^8^ and ≤13.4 × 10^8^ in the leukapheresis product five of six patients (83%) experienced a partial remission, while one (17%) patient achieved complete remission.

### 3.8. Comparison of Leukapheresis Products from Patients by Remission Status and Healthy Donors

**Figure 6:**
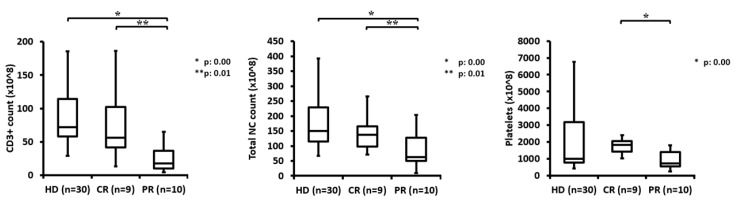
Comparison of leukapheresis products from healthy donors and patients by remission status. Total NC count, CD3+ count, and platelets are compared between healthy donors (*n* = 30) and patients receiving axicabtagene ciloleucel, sorted by response.

Leukapheresis product parameters of 21 patients receiving axicabtagene ciloleucel, adjusted to remission status, were compared to 30 HDs leukapheresis products (Figure 6). 

Patients achieving a complete remission had a significantly higher median CD3+ T cell count of 5.6 × 10^9^ (1.3 - 18.6 × 10^9^) and TNC count of 13.7 × 10^9^ (7.2 - 26.5 × 10^9^) than patients with partial response (median CD3+ T cell count of 1.7 × 10^9^ (0.4 - 6.5 × 10^9^), TNC count 6.2 × 10^9^ (0.9 - 20.3 × 10^9^)) and patients with progressive disease (median CD3+ T cell count of 4.1 × 10^9^ (1.9 - 6.2 × 10^9^). Even so, the CD3+ T cell count and TNC count differed between the patient groups, the lines of pretreatment between patients with CR and patients with PR were comparable (median of 4 (2-7) vs. 4 (2-8)). For patients with SD/MR or PD the lines of pretreatment seem to be higher with a median of 5 (4-6), however the data are limited and have to be evaluated in further studies. The main impact on the patient outcome was the disease progression. Patients with a rapid progressive disease requiring a bridging therapy between leukapheresis and CAR-T cell therapy had a worse outcome (Figure 4B). Additionally, an important topic of this analysis was the comparison of leukapheresis products from healthy donors to patients with regard to CAR-T cell production off the shelf. No significant difference was observed to leukapheresis products from patients achieving complete remission. In contrast, a significant difference could be seen between the leukapheresis products of these healthy donors (median CD3+ T cell count of 7.2 × 10^9^ (2.9 - 18.6 × 10^9^), TNC count of 14.9 × 10^9^ (6.6 - 39.3 × 10^9^) and platelet count of 98.7 × 10^9^ (41.8 - 755.1 × 10^9^)), and leukapheresis products from patients achieving only partial remission or suffering from progressive disease. 

Further analysis yielded the following cut-off scores for apheresis products: 1.2 × 10^9^ CD3+ T cell count, 2.8 × 10^9^ TNC count, and 51.0 × 10^9^ platelet count. A total of 90% of all patients with apheresis products beyond these three cut-off criteria achieved partial or even complete response.

## 4. Discussion

Efficient leukapheresis providing a sufficient amount of T lymphocytes is a critical step in the manufacturing process of CAR-T cells. Stem cell donation centers have already a longstanding experience with donor lymphocyte infusions. In HDs, a large number of T lymphocytes can be collected with an unstimulated leukapheresis (Table 3 and Table 4). However, only little data is available on T lymphocyte collections in patients intended for CAR-T cell therapy [6,13]. Moreover, patients currently evaluated for CAR-T cell treatment suffer from relapsed/refractory disease and have therefore been treated with at least two lines of chemotherapy prior to the intended CAR-T cell treatment. Depending on the remission status, the biology and the previous course of the underlying disease, a significant number of patients—almost half in our investigation (44%)—require some kind of bridging therapy until infusion of the CAR-T cell product. In addition, clinical evaluation of the patient, time needed for tumor board decision, treatment schedule of T cell impairing medications, the apheresis slot, the CAR-T cell production slot, and the requirement of hospital admittance for CAR-T cell infusion have to be taken into consideration posing a tremendous challenge on the process of timing, preparation, and execution of the leukapheresis procedure.

To optimize the workflow between the patient’ first visit with subsequent tumorboard decision and the T lymphocyte collection by leukapheresis, an adequate check-up of the patient according to standard operating procedures (SOP) is mandatory. Figure 2 displays the workup for potential CAR-T cell patients in our department, designed in analogy to the well-established procedures for patients receiving an autologous or allogeneic hematopoietic stem cell transplantation. It comprises a clinical check-up with a detailed patient history and clinical examination including ECG, ECHO, lung function test, CT or ultrasound where applicable, blood counts with electrolytes, values for liver, kidney, thyroid function, clotting levels, inflammation markers, screening for infectious disease markers as HBV, HCV, HEV, HIV, and *Treponema pallidum* antibodies, as well as an assessment of venous access and finally an informed consent as a prerequisite for the leukapheresis and CAR-T cell therapy.

As part of the initial check-up, detailed documentation of previous chemotherapy treatment in particular of T cell impairing medications is essential, because little is known about the influence of antecedent chemotherapy on CAR-T cell generation. Many chemotherapeutics such as alkylating agents have a negative impact on T cells [14]. The same applies to high-dose steroid therapy, which leads to a rapid depletion of circulating lymphocytes [15,16,17]. However, low-to-moderate doses of steroids might lead to a slight reduction of lymphocytes with normalization by 24-48 h after discontinuation of treatment [16,18,19].

**Figure 7:**
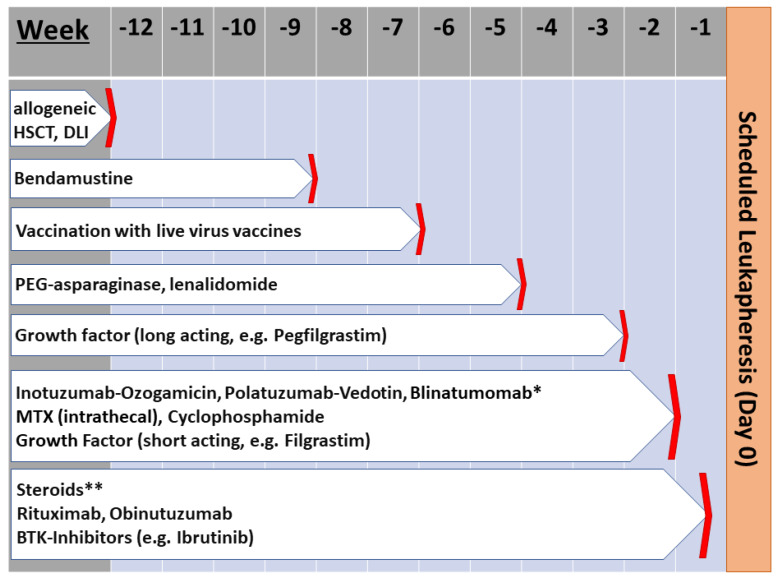
Stopping rules for ongoing therapies prior to apheresis (HD-CAR-1). Guidelines established for the HD-CAR-1 study at the University Hospital Heidelberg. * First cycle application only. For additional cycles 10–14 days are recommended. ** Low-to-moderate steroid doses only. BTK: bruton kinase, DLI: donor lymphocyte infusion, HSCT: hematopoietic stem cell transplantation, MTX: methotrexate, PEG: pegulated.

For commercially available CAR-T cell products, the initiation of a washout period prior to leukapheresis is at the discretion of the treating physician. However, there are some recommendations regarding the discontinuation of previous therapies based on their clinical studies [1,2]. Within our HD-CAR-1 clinical trial, recommendations for discontinuation of prior treatment regarding this were set as shown in Figure 7. 

G-CSF (granulocyte colony stimulating factor) induces stem cell mobilization. Stem cells in the leukapheresis product pose the risk of malignant transformation during the process of genetic modification by viral transduction. Therefore G-CSF should be stopped prior to leukapheresis [20,21,22]. Vaccinations with live vaccines should be stopped 6 weeks prior to leukapheresis to prevent virus particles in the CAR-T cell product from being transmitted to the immunosuppressed patient potentially causing a serious viral infection [23]. Furthermore, many standard therapies such as PEG-asparaginase for ALL, the alkylating agent Bendamustin, and the immunomodulator lenalidomide have a profound negative impact on T cell function/proliferation and should not be given within 4 weeks prior to leukapheresis [24].

Antibody-drug conjugates such as Polatuzumab Vedotin, a humanized CD79b antibody conjugate with a potent mitosis inhibitor monomethyl auristatin (Plivy^®^, Roche), and Inotuzumab Ozogamicin, a humanized CD22 antibody linked to the cytotoxic antibiotic calicheamicin (Besponsa^®^, Pfizer/Wyeth), combine the capabilities of tumor cell targeting and tumor cell elimination [25,26]. The goal of these conjugates is to gain access to the tumor cells with direct cytotoxin release and tumor cell kill without harming the healthy cells and limiting the systemic exposure to the drug. In a mechanism based pharmacokinetic model the tumor drug concentration of calicheamicin after a single dose of 1.8 mg/m^2^ was predicted to be around 0.1 ng/mL after 7 days [27]. Therefore, a wash-out period of at least 7 days should be observed. In addition, drug-related leuko- and lymphocytopenia should be kept in mind with a close monitoring of the patient.

Bispecific T cell engager (BiTE) as blinatumomab are a class of therapeutic antibodies that consist of two single-chain variable fragments (scFv) simultaneously targeting tumor antigens (e.g., CD19) on cancer cells and CD3 of the T cell receptor complex, hereby bringing target and effector cells into close proximity and activating the cytotoxic function of T cells. The half-life of blinatumomab is only about 1.25 h, therefore it has to be administered by continuous intravenous infusion in repeated 4-week cycles. Serum levels of blinatumomab decline quickly after discontinuation of infusion [28]. In addition to expected B cell depletion, blinatumomab infusion also leads to an initial decline of the T cell count which usually recovers after a few days and further develops into T cell expansion. Constant exposure of patients to the BiTE blinatumomab did not lead to signs of T cell anergy [28]. Nevertheless, a wash-out period of at least 1 week after the first cycle and 2 weeks after two and more cycles is recommended.

Rather positive effects on T cells have been attributed to the Bruton’s tyrosine kinase (BTK) inhibitors. BTK-inhibitors like ibrutinib have shown not to negatively impair the leukapheresis or function of CAR-T cells in CLL patients. Fraietta et al. showed that ibrutinib selectively inhibited Th2 responses and decreased the expression of exhaustion marker PD-1 on T cells. [29]. Due to these positive effects, ibrutinib can be administered until shortly before leukapheresis and simultaneous use of ibrutinib with CAR-T cell therapy is currently under investigation [29,30,31].

Due to the frequently low leukocyte and lymphocyte count at the time of leukapheresis, the lymphocyte count prior to apheresis and CD3+ T cell yield in the product were matched to the remission outcome (Figure 5). A total of 80% of patients with lymphocyte counts below 0.6/nL in the peripheral blood and subsequent CD3+ T cell yield below 20 ×10^8^ in the apheresis product achieved at least a partial remission after CAR-T cell infusion. Thus, it can be concluded that even in patients with low lymphocyte counts leukapheresis is feasible and collected T cells can be transformed into a functional CAR-T cell product. In addition, in a comparison of leukapheresis products from 21 patients receiving axicabtagene ciloleucel, patients with leukapheresis products containing more than 70 × 10^8^ CD3+ T cells and 205 × 10^8^ TNCs reached a complete remission. Additionally, patients achieving complete remission had significantly higher CD3+ T cell and TNC counts than patients with partial remission (see Section 3.8). In contrast, CD3+ T cell and TNC counts of these patients with CR were comparable to leukapheresis products of healthy donors (Figure 6). This interesting finding however is based on a small sample size of only 25 patients evaluated for remission and should be further evaluated in clinical practice with regard to autologous or off the shelf CAR-T cell production.

Leukapheresis has long been practiced in an out-patient setting without any serious complications [32]. For the heavily pretreated and debilitated patient population however a detailed medical review is essential to detect potential causes of complications early on. Common comorbidities of those heavily pretreated patients are chronic renal insufficiency with or without electrolyte disturbance, progressive disease with bone involvement leading to hypercalcemia, disposition to infections or anticoagulation therapy due to thrombotic events. In addition, immediate admittance should be considered in case of disease progression.

Patients with progressing disease receive a bridging therapy and therefore are often in a leukopenic situation or suffer from other disease- and/or treatment-related complications. Nevertheless, even in this patient population leukapheresis is safe and frequently results in the collection of sufficient numbers of CD3+ lymphocytes for the manufacture of CAR-T cells [33,34]. One single hospitalization was necessary due to hypercalcemia in the course of disease progression. The aim is to standardize the pre-leukapheresis check-up and the cell collection via leukapheresis to prevent possible collection failures or loss of time. Figure 3 shows an algorithm addressing the clinical check-up, blood values, and infectious disease markers with the respective criteria leading to a cancellation or delayed collection.

Moreover, patients with progressive disease, especially patients suffering from acute lymphcytic leukemia, might present with a leukemic phase. This might pose a challenge for the manufacturing center. Ruella et al. recently published an unintentional CAR-transduction of a leukemic B cell during CAR-T manufacturing. This resulted in the relapse, progressive leukemia and eventually death of a patient with ALL after treatment with CD19-targeting CAR-T cells [35]. In the leukapheresis concentrate of this patient the contamination with leukemic cells was high however also several other patients had even higher B cell frequencies without developing a CAR+ leukemia. This is a rare event, as this is the only case with a CAR+ leukemia relapse in patients suffering from ALL reported worldwide. No such case has been hitherto reported for patients with lymphoma who very rarely present in a leukemic state of the lymphoma.

## 5. Conclusions

Leukapheresis was feasible in all patients and could be performed in an out-patient setting. In patients with a low WBC (white blood cell) count of 1–3/nL and an absolute lymphocyte count of ≤1/nL, a minimum of 12–15 L blood volume should be processed to harvest a sufficient number of lymphocytes for CAR-T cell production. Furthermore, discontinuation of T cell impairing therapies should be adjusted to the planned leukapheresis date. Therefore, standardized procedures based on an algorithm are mandatory to ensure efficient collection.

## Figures and Tables

**Table 1:** Overview of the different chimeric antigen receptor (CAR) T cell products and requirements by manufacture.

Therapeutic Framework/Indication			Apheresis Procedure		Leukapheresis Product			Application		
Commercial Product (C)/Clinical Trial Product (T)		Indication	HK (%)	Anticoagulant	TNC × 10^8^	CD3+ × 10^8^	CD3+ of TNC (%)	Lympho-Depleting Chemotherapy	CAR-T Cell Dose	Time Thawing—Transfusion
**C**	**Axicabtagen Ciloleucel**	r/r DLBCL, PMBCL	nr	nr	nr	nr	nr	F 30 mg/m^2^	0.4 - 2 × 10^8^	3 h
	(Kite)							C 250 mg/m^2^		
	**Tisagen-lecleucel**	ALL < 26 yo, r/r DLBCL	4	ACD-A or ACD-A plus Heparin	≥20	≥10	≥3	F 25 mg/m^2^	ALL: 2.5 × 108	30 min
	(Novartis)							C 250 mg/m^2^	DLBCL: 0.6 - 6 × 108	
**T**	**HD-CAR-1** (Heidelberg)	r/r ALL, NHL, ped r/r ALL	nr	nr	nr	nr	nr	F 30 mg/m^2^	Dose I: 1 × 10^6^/m^2^ Dose II: 5 × 10^6^/m^2^ Dose III: 20 × 10^6^/m^2^	2 h
								C 500 mg/m^2^		

DLBCL = diffuse large B-cell lymphoma, PMBCL = primary mediastinal B-cell lymphoma, ALL = acute lymphoblastic leukemia, NHL = Non-Hodgkin’s lymphoma, r/r = relapsed or refractory, HK = hematocrit, TNC = total nucleated cells, F = fludarabine, C = cyclophosphamide, nr = no requirement.

**Table 2:** Baseline characteristics of patients.

Characteristics of All Patients	All Patients	Male	Female
	*n* = 41	*n* = 29	*n* = 12
**Age** (years), median (range)	56 (20–72)	56 (20–70)	56 (20–72)
**Weight** (kilogram), median (range)	79 (47–147)	82 (53–147)	60 (47–85)
**Height** (centimeters), median (range)	176 (150–197)	180 (163–197)	164 (150–171)
**Total blood volume** (L), median (range)	5.1 (3.2–7.6)	5.4 (3.9–7.6)	3.6 (3.2–4.6)
**Characteristics (Yescarta^®^ group)**	**25 Patients receiving leukapheresis**		
**Patient gender**	Male *n* = 19 (76%), Female *n* = 6 (24%)		
**Age** (years), median (range)	54 (20–68)		
**Disease**	DLBCL *n* = 24 (96%), PMBCL *n* = 1 (4%)		
**Prior therapy lines** median (range)	5 (2–8)		
**Best response**	CR (36%), PR (40%), SD/MR (4%), PD (8%), NE (12%)		

DLBCL = diffuse large B-cell lymphoma, PMBCL = primary mediastinal B-cell lymphoma, CR = complete remission, PR = Partial remission, SD/MR = stable disease/mixed response, PD = progressive disease, NE = not evaluable.

**Table 3:** Leukapheresis characteristics.

Characteristics	Healthy Donors	Lymphoma	CLL	ALL
	*n* = 30	*n* = 32	*n* = 2	*n* = 6
**Leukapheresis duration**	n.a.	239 (120–300)	213 (180–245)	271 (185–300)
(minutes), median (range)				
**Processed blood volume**	10.5 (5.8–15.0)	12.0 (5.8–15.0)	9.5 (9.0–10.0)	9.0 (6.4–15.0)
(L), median (range)				
**Times TBV processed**	2.1 (1.7–3.1)	2.5 (1.2*–3.9)	1.6 (1.5–1.7)	2.3 (1.3–2.7)
median (range)				
**Neutrophil count prior**	4.0 (2.8–5.7)	4.1 (2.1–18.7)	3.0 (2.9–3.1)	2.9 (1.4–3.9)
/nL, median (range)				
**Lymphocytes count prior**	1.3 (0.7–1.6)	0.7 (0.2–3.4)	1.0 (0.4–1.6)	0.4 (0.1–1.5)
/nL, median (range)				

* For the HD-CAR-1 study a TBV processed ≥ 1.2 was sufficient. CLL = chronic lymphocytic leukemia, ALL = acute lymphoblastic leukemia, TBV = total blood volume, n.a. = not assessed.

**Table 4:** Leukapheresis product characteristics.

Characteristics	Healthy Donors	Lymphoma	CLL	ALL
	*n* = 30	*n* = 32	*n* = 2	*n* = 6
**Total nucleated cells**	149.0 (66.4–392.7)	100.4 (9.3–340.5)	79.6 (78.3–80.8)	62.5 (19.7–156.0)
(×10^8^), median (range)				
**Total CD3+ cell count**	72.0 (4.1–185.9)	41.0 (4.2–231.8)	36.7 (23.5–49.8)	26.0 (4.0–68.0)
(×10^8^), median (range)				
**Volume**	176.6 (106.0–268.7)	238.0 (136.0–310.0)	223.0 (190.0–255.0)	235.5 (188.0–289.0)
(mL), median (range)				
**Hematocrit**	3.9 (1.8–7.4)	2.6 (1.3–7.4)	2.2 (2.1–2.2)	2.5 (1.1–3.3)
(%), median (range)				
**Monocytes**	n.a.	24.7 (8.1–53.8)	27.3 (6.9–47.8)	14.7 (6.2–33.0)
(%), median (range)				
**Platelets**	987.0 (418.0–7551.0)	1088.0 (147.0–3120.0)	1004.5 (966.0–1043.0)	615.5 (170.0–1310.0)
(×10^8^), median (range)				
**Viability**	99.8 (99.6–100)	99.9 (99.6–100)	99.8 (99.8–99.8)	99.8 (99.6–99.9)
(%), median (range)				

CLL = chronic lymphocytic leukemia, ALL = acute lymphoblastic leukemia, TBV = total blood volume, n.a. = not assessed.

**Table 5:** Overview of patient information and course of therapy.

Number of Patients	Sex	Age	Diagnosis	Pretreatments	Disease Status before Leuka-Pheresis	Last Treatment (<12 Weeks before Leukapheresis)	Pre-Apheresis TNC in Peripheral Blood	Pre-Apheresis Lymphocyte Count	Apheresis Concentrate TNC Count	Bridging Therapy Prior CAR-T Cell Therapy	Response to CAR-T Cell Therapy
	(male/female)	median (range)		median (range)		(percentage; weeks prior leukapheresis)	median (range), /nL	median (range), ×10^8^	median (range), ×10^8^	**w/w/o (%)**	
**25** patients (with 23 patients receiving axicabtagen ciloleucel) *^,^°	76%/24%	54 (20–68)	DLBCL (96%) PMBCL (4%)	5 (2–8)	PR (4%), SD/MR (8%), PD (84%), NE (4%)	Ibr (4%), Rituximab (20%), R-DHAO/P (12%), R-ICE (8%), R-B (4%), Len (4%), GDP (4%), GemOx (8%), Dexa (4%), Ino (4%), Brent (8%), Pola (4%), st (4%), NT (12%)	4.3 (2.2–18.7)	0.6 (0.2–3.4)	100 (9–341)	57%/43%	CR (36%) PR (40%) SD/MR (4%) PD (8%) NE (12%)
**Four** patients receiving second apheresis *	100%/0%	51 (27–63)	DLBCL (100%)	4 (2–5): R-CHOP (100%), R-DHAO/P (50%), (R-) ICE (50%), R-Pola (25%), allogeneic SCT (25%), Nivolumab (25%)	PD (100%)	First leukapheresis: P1: R-DHAP (5 wks) P2: R-DHAP (8 wks) P3: Rituximab (1 wk) P4: NT	First leukaph.: 5.8 (4.1–8.6)	First leukaph.: 0.5 (0.4–0.7)	First leukaph.: 128 (75–341)	67%/33% bridging therapy: Ibr, R-B, Pola-BR	PR (33%) PD (33%) NE (33%)
						Second leukapheresis: P1: R-DHAP (8 wks) P2: Dexa (2 wks) P3: Rituximab (3 wks) P4: NT	Second leukaph.: 4.3 (3.5–5.4)	Second leukaph.: 0.6 (0.4–0.9)	Second leukaph.: 104 (68–169)		
**One** patient CAR-T cell production failed *	male	63	DLBCL	3: R-CHOP, R-DHAP, R-B	PD	R-DHAP (8 wks prior first leukapheresis)	First leukaph.: 8.6	First leukaph.: 0.4	First leukaph.: 75	-	-
						Dexa (2 wks prior second leukapheresis)	Second leukaph.: 4.3	Second leukaph.: 0.9	Second leukaph.: 86		
**One** patient deceased prior to infusion °	male	52	DLBCL	3: R-CHOP, R-DHAP, R-ICE	PD	R-ICE (9 wks prior)	18.7	2.2	317	-	-

Percentages (%) denote how many patients received a certain treatment or achieve the stated remission status. *^,^° = patients are listed in the main group as well as in subgroups. DLBCL = diffuse large B-cell lymphoma, PMBCL = primary mediastinal B-cell lymphoma, MCL = mantle cell lymphoma, FL = follicular lymphoma, CLL = chronic lymphocytic leukemia, ALL = acute lymphoblastic leukemia, TNC = total nucleated cells, NE = not evaluable, CR = complete remission, PR = partial remission, SD/MR = stable disease/mixed response, PD = progressive disease, SCT = stem-cell transplantation, Ibr = ibrutinib, R-DHAO/P = rituximab/cytarabine/dexamethasone/platin (cis- or oxali-), R-B = rituximab/bendamustine, GDP = gemcitabine/dexamethasone/cisplatin, Len = lenalidomide, Pola-(B)R = polatuzumab with rituximab (and bendamustine), GemOx = gemcitabine/oxaliplatin, Dexa = dexamethasone, Ino = inotuzumab, Brent = brentuximab, Pola = polatuzumab, st = study, NT = no treatment, P = patient, wk(s) = week(s).
